# Automated measurement of heterogeneity in CT images of healthy and diseased rat lungs using variogram analysis of an octree decomposition

**DOI:** 10.1186/1471-2342-14-1

**Published:** 2014-01-06

**Authors:** Richard E Jacob, James P Carson

**Affiliations:** 1 Biological Sciences Division, Pacific Northwest National Laboratory, 902 Battelle Blvd., Richland, WA 99352, USA

**Keywords:** Lung imaging, Disease detection, COPD, Emphysema, Pulmonary, Octree, Variogram

## Abstract

**Background:**

Assessing heterogeneity in lung images can be an important diagnosis tool. We present a novel and objective method for assessing lung damage in a rat model of emphysema. We combined a three-dimensional (3D) computer graphics method–octree decomposition–with a geostatistics-based approach for assessing spatial relationships–the variogram–to evaluate disease in 3D computed tomography (CT) image volumes.

**Methods:**

Male, Sprague-Dawley rats were dosed intratracheally with saline (control), or with elastase dissolved in saline to either the whole lung (for mild, global disease) or a single lobe (for severe, local disease). Gated 3D micro-CT images were acquired on the lungs of all rats at end expiration. Images were masked, and octree decomposition was performed on the images to reduce the lungs to homogeneous blocks of 2 × 2 × 2, 4 × 4 × 4, and 8 × 8 × 8 voxels. To focus on lung parenchyma, small blocks were ignored because they primarily defined boundaries and vascular features, and the spatial variance between all pairs of the 8 × 8 × 8 blocks was calculated as the square of the difference of signal intensity. Variograms–graphs of distance vs. variance–were constructed, and results of a least-squares-fit were compared. The robustness of the approach was tested on images prepared with various filtering protocols. Statistical assessment of the similarity of the three control rats was made with a Kruskal-Wallis rank sum test. A Mann-Whitney-Wilcoxon rank sum test was used to measure statistical distinction between individuals. For comparison with the variogram results, the coefficient of variation and the emphysema index were also calculated for all rats.

**Results:**

Variogram analysis showed that the control rats were statistically indistinct (p = 0.12), but there were significant differences between control, mild global disease, and severe local disease groups (p < 0.0001). A heterogeneity index was calculated to describe the difference of an individual variogram from the control average. This metric also showed clear separation between dose groups. The coefficient of variation and the emphysema index, on the other hand, did not separate groups.

**Conclusion:**

These results suggest the octree decomposition and variogram analysis approach may be a rapid, non-subjective, and sensitive imaging-based biomarker for characterizing lung disease.

## Background

Emphysema is an obstructive pulmonary disease that results in airway expansion and tissue destruction. Early and accurate detection of emphysema is important for disease management and improved patient outcomes [1]. Emphysema typically results in heterogeneous air trapping and increased ventilation-perfusion inequality. Thus, signatures of tissue heterogeneity or regional air trapping may facilitate earlier and/or more accurate diagnoses of emphysema [2,3].

It has been shown in many studies that CT is a reproducible and predictive modality for diagnosing and assessing emphysema [2,4-7]. The most common way to quantify moderate to severe emphysema from CT images is to measure the emphysema index: the percentage of voxels below a preset Hounsfield Unit (HU) threshold. However, this index may not detect subtle or early onset emphysema [7], or, conversely, it may identify “false positive” regions that appear emphysematous in asymptomatic healthy young subjects with no smoking history [8]. In addition, threshold values vary between studies, typically within a range of-900 to-980 HU, with factors other than disease influencing CT densitometry [2,4,9,10].

An approach to assessing heterogeneity that has been applied to myriad types of medical images is fractal analysis [11]. This approach exploits the scale-independent nature of fractals in systems, such as lung vasculature, whose variation in form or regularity is thought to be similar through different degrees of magnification [12]. Typically, a region (or regions) of interest (ROI) is selected, which is then evaluated for a mean value and/or repeatedly subdivided to relate differences in signal intensity across space and across ROI size. If the relationship is characterized by a power law, then the exponent, or fractal dimension, is taken as an indication of complexity, texture, or heterogeneity [13-15]. However, this method is based on an a priori assumption that power law relationship exists and that a fractal dimension can be determined. Furthermore, because this approach requires ROI selection, it is often subjective and can result in omission of large sections of the lung [13,16].

A promising new approach, detailed in recent work by Subramaniam et al. [17], demonstrates the use of analysis of CT image slices using a quadtree decomposition. This approach iteratively divides a 2D image into increasingly small squares. Division occurs whenever the range of intensities within the quadrant are above a pre-defined threshold. In this way, regions with homogenous intensity are characterized by larger squares. Their work measures heterogeneity by counting the number of squares-per-area in the lung or in a local region of the lung.

We propose to extend the quadtree decomposition to 3D by using octrees. In this way, the entire lung CT image volume is used. An octree iteratively divides a cubic volume into eight evenly sized cubes, or octants. The octree method is well-developed and is used, for example, in 3D mesh generation and computer graphics [18,19], medical image registration [20], and finite element meshing in CT images [21]. The advantages of the octree here is that it facilitates a speed-up in 3D lung tissue analysis by non-subjectively subdividing the lungs into homogeneous regions, thereby allowing for focus on parenchyma by reducing partial volume averaging and eliminating edges [22].

To incorporate spatial information across the lung, we propose to couple the octree image decomposition approach with variogram analysis. Variograms are a spatio-statistical approach for measuring spatial variability by comparing sample value variances to the distance of separation [23,24]. Variograms are well-established in geostatistics, but have also recently attracted interest in biomedical applications, such as characterizing magnetic resonance images of white matter [25]. In this pilot study using our approach, we are able to significantly differentiate from each other the three groups of elastase-dosed rats: control, distributed mild emphysematous disease, and region-specific severe disease.

## Methods

### Disease model

An elastase-induced model of emphysema was used in this study. Nine male Sprague-Dawley rats with an average weight of 212 ± 11 g were orally intubated and dosed intratracheally with: 250 U/kg elastase dissolved in 200 μL saline to the whole lung (n = 3), or 50 U/kg elastase in 200 μL saline to a single lobe (n = 3), or 200 μL saline as a control (n = 3). Dosing levels were based on our previous work in which emphysematous changes were detected using ^3^He diffusion MRI and histology [26]. All animal use was approved by the Institutional Animal Care and Use Committee at Pacific Northwest National Laboratory.

### CT imaging

Three weeks following dosing, the rats were imaged using micro-CT. At this time, rats weighed 357 ± 10 g. The imaging procedure is described in detail in [27]. Briefly, rats were anesthetized, intubated, and mechanically ventilated at 1 Hz with 40% inhale and 60% exhale durations. Peak inhalation pressure was ~8 cmH_2_O, and no peak end expiratory pressure was used so that images could be acquired at functional residual capacity. Anesthesia was maintained by providing 3-4% isoflurane in air (30% O_2_, balance N_2_). Sigh breaths were delivered periodically to maintain lung recruitment. A respiratory-gated GE eXplore 120 micro-CT scanner was employed with the following settings: 100 kV peak voltage, 50 mA tube current, 16 ms exposure time, and 360 projections with 1 degree angular steps. Images were reconstructed with supplied software to 150 μm isotropic resolution. Total imaging time was about 90 minutes due to the collection of multiple images throughout the breathing cycle; however, only the images acquired at full exhalation were used for the analyses herein. Post-mortem histology and tissue analysis were not possible due to *in situ* lung casting for other research purposes (i.e. supplying airway tree geometries for computational fluid dynamics models [28,29]).

### Image preparation

A lung mask image was semi-automatically generated from the above-mentioned reconstructed images using ImageJ [30] and the ImageJ 3D Toolkit plug-in [31]. Starting from a seed-point inside the lung, a 3D connected threshold was applied with a threshold value empirically selected to exclude major vasculature and all external tissue, so that only lung tissue would be included in analysis. Mask boundaries were smoothed using the Region Dilate and Region Erode functions in succession. Applying the mask to the reconstructed images, all background was thus assigned an intensity value of 0, and all unmasked lung tissue retained original HU values. A five pixel diameter 3D median filter was then applied in order to reduce noise while contributing minimal blurring. Finally, the image canvas size was increased to 512 × 512 × 512 by zero-filling in each direction. We note that zero-filling to a power of 2 served to conveniently restrict all octants in the octree decomposition to isotropic cubes.

### Octree decomposition

**Figure 1:**
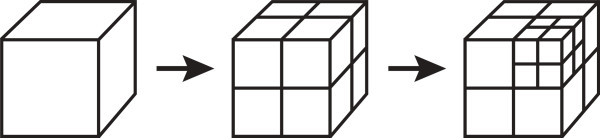
Octree decomposition.

An automated octree decomposition was performed by iteratively subdividing an image, with each division producing eight evenly sized octants (e.g., the initial 512 × 512 × 512 image was decomposed into eight 256 × 256 × 256 octant regions, and so on; see Figure 1) [32]. After each division, the maximum, minimum, mean, and standard deviation of the signal intensity were calculated for each octant region. Then, iterative octant subdivision of a region occurred if either the standard deviation of that region exceeded a user-defined threshold, or if the region contained at least one voxel–but not all voxels–with intensity equal to the background signal (i.e. 0). However, regions reaching a minimum size of 2 × 2 × 2 were not subdivided. At the end of the decomposition, all boxes with a mean HU value ≠ 0 and dimensions greater than 2 × 2 × 2 represented portions of lung with relatively uniform HU values. Following decomposition, results were binned according to HU and box size for histogram display. Boxes containing at least one 0 were not included in the binning. Octree decomposition was implemented in Python and executed on a MacPro model 3.1. We note that we have modified our Python code to integrate ImageJ to minimize user interaction, although that was done subsequent to this work. The only interactive user-initiation required is the manual selection of a seed-point for the 3D connected threshold.

There are multiple approaches to selecting the aforementioned user-defined threshold criteria. For example, in [17] boxes were subdivided if the HU range in the box exceeded 10% of the range of values in the entire image. Because our rats had different ranges of HU values depending on disease severity, we chose to use the standard deviation as the threshold criterion, as it does not require normalization. We describe how the threshold range was determined in Section Threshold range.

### Variogram analysis

The purpose of using variograms in this context was to determine the spatial relationships between regions of the lung that are relatively homogeneous. The octree decomposition isolated regions of relatively high homogeneity; therefore, each resulting box was treated as a homogeneous “voxel” using the mean value of the box as the voxel intensity. Furthermore, it was important to use octree boxes that were all the same size. The largest box sizes that resulted from the octree decompositions were mostly 8 × 8 × 8 with a few 16 × 16 × 16 boxes. In order to ensure a sufficient number of boxes that were distributed throughout the lung and to enhance statistical power [25], each 16 × 16 × 16 box was further broken down into 8 × 8 × 8 boxes, and all 8 × 8 × 8 boxes were used in the variogram analysis. This approach assumed that emphysematous disease was manifest on a scale greater than an 8 × 8 × 8 box, which for these images is 1.2 mm on a side–this is consistent with what we observed in this disease model in previous work [26]. Importantly, excluding the smaller boxes largely eliminated vasculature, conducting airways, boundaries, and other features that had high spatial variability [22].

The calculation of the variance (or semi-variance) γ(*d*) of the differences in signal intensity *I* is described in detail in many geostatistics texts, such as [23,24], and is given by:

(1) $$y\left(d\right)=-\frac{1}{2N\left(d\right)}{\sum_{i}^{N\left(d\right)}\left[I\left(x_{i}\right)-I\left(x_{i}-d\right)\right]}^{2}$$

where *d* is the distance between voxel centroids, *x* is the voxel location in 3D, and *N(d)* is the number of voxel pairs for a given *d*. γ(*d*) is calculated for all values of *d*. For an image with *n* voxels, the total number of voxel pairs (for all existing values of *d*) is given by

(2) $$N=n\left(n-1\right)/2.$$

We then calculated the average semi-variance at each distance. Results were plotted on a distance vs. variance graph, or variogram, which graphically represents the spatial dissimilarity within the image.

The purpose of the variograms was to characterize spatial relationships between boxes of relatively high homogeneity that were dispersed throughout the lung. However, as described by [25] in work with brain images, increased distances between voxels, or octants, lead to decreased likelihood that the voxels are related in any way. This is especially true in the lung, which is composed of largely independent lobes (five in the rat). Moreover, the spatial relationship between parenchymal signal intensity in different lobes is, in many contexts, most accurately described by a unique path up and back down the airway tree, potentially covering 20 or more orders of branching [33]. The spatial distances are analogous to the “regionalized variable” in the geological context of mineral distribution. Indeed, the regionalized variable, or the maximum distance *d*_*max*_ over which the variogram is expected to be reliable, is estimated to be one half of the diameter of a region [25], while beyond *d*_*max*_ any relationships between distance and variance are expected to be random. Therefore, we limited our variogram analysis to a region that represented half of the average dimension of the left lobe, which is typically the largest lobe in the rat. This assumes that the disease varies slowly compared to the box size but rapidly compared to the lobe size. Using the 3D images and a lung cast, *d*_*max*_ was measured to be ≈ 8 mm (53 pixels) in the healthy rats, which was rounded up to the equivalent of seven face-bordering 8 × 8 × 8 boxes, or 56 pixels.

### Octree decomposition tests

We performed the following evaluations of the appropriateness and robustness of the octree and variogram approach.

#### Threshold range

The octree decomposition compared the standard deviation of the lung signal within a box to a threshold range to determine whether the box should be subdivided. The optimal threshold range was determined semi-empirically. As a starting point, we calculated the typical standard deviation of the lung tissue of the three control rats. This was accomplished by producing a histogram of each masked image and then fitting the main lung peak to a Gaussian curve. The mean of the control rats’ standard deviations (σ) was set as the initial threshold range. We then tested the effects of thresholds that were σ/3, 2σ/3, 4σ/3, and 2σ by rerunning the decomposition on the same images. We evaluated the effectiveness of each threshold range at grouping the control rats and at distinguishing the full-lung-dose rats from the control group in the variograms.

#### Image filtering

We examined the effects of image filtering by generating variograms from unfiltered images, images filtered with a five pixel diameter 3D median filter, images filtered with a 3D Gaussian blur of radius = 2, and images filtered with a 3D Gaussian blur of radius = 4. Filters were applied in ImageJ prior to applying the mask. The number of octree boxes and differences in the variograms were compared. Adjustments to the threshold range were made for each filter test based on the standard deviation of the filtered image.

#### Image translation

Rat positioning during imaging can vary from animal to animal, and the octree decomposition should be independent of this. Therefore, we tested the effects of image translation on the variogram results. For the variogram analysis, the largest unit box retained after decomposition was 8 × 8 × 8; therefore, a shift in the image by 8 pixels in any (or all) dimensions should result in no change to the resulting variogram, since the original image had isotropic dimensions of 2^n^. Conversely, maximum change would occur from a four-pixel shift. To test the significance of the effects of translation, the image of one rat was shifted in x, y, and z by four pixels and by eight pixels using ImageJ, and variogram results were compared to those of the original image.

#### Image rotation

Animal positioning can also have an apparent effect on image rotation, and octree decomposition should be insensitive to this. Using the same rat image as used in the translation test, we imitated an arbitrary 3D rotation by rotating the image π/4 radians about the x, y, and z axes using ImageJ. Results were compared to the original image and to the results of the translation test.

#### Image downsampling

To demonstrate the increased value of the combined octree and threshold approach for establishing the 8 × 8 × 8 boxes versus simply downsampling the image, we generated variograms using standard image downsampling as a comparison. For this, we applied a bilinear downsampling to the 512 × 512 × 512 image, creating a 64 × 64 × 64 image–each voxel the equivalent size of an 8 × 8 × 8 octree box. Then we generated variograms utilizing the intensity value of every voxel in the 64 × 64 × 64 image (excluding the background voxels, again defined as those voxels with intensities equal to zero).

### Emphysema index, coefficient of variation, and heterogeneity score

The percentage of lung below a HU threshold value, or emphysema index, was calculated for each rat to compare this conventional measurement of disease severity with the variogram results. Because there is no established HU threshold level in rat models of emphysema, we chose to count the percentage of voxels with HU values below two standard deviations from the control-group mean. This level was determined to be−717 HU. Calculations were made on the same masked images used for octree decomposition.

The CoV was calculated for each rat by fitting a histogram of the masked lung images to a Gaussian curve and taking the ratio of the standard deviation to the mean.

For comparison, we defined a new metric, the heterogeneity score (Δ), as the average difference between the mean variance of the control group and the spatial variance of each individual rat, in the range *d* ≤ *d*_*max*_.

### Statistical analysis

Comparisons of variograms of the three control group rats were made using the Kruskal-Wallis (KW) rank sum test with a null hypothesis of α = 0.05. This indicated whether there were significant differences within the group. For pairwise comparisons, a Mann-Whitney-Wilcoxon (MWW) rank sum test was employed, also with a null hypothesis of α = 0.05.

## Results

### CT images

**Figure 2:**
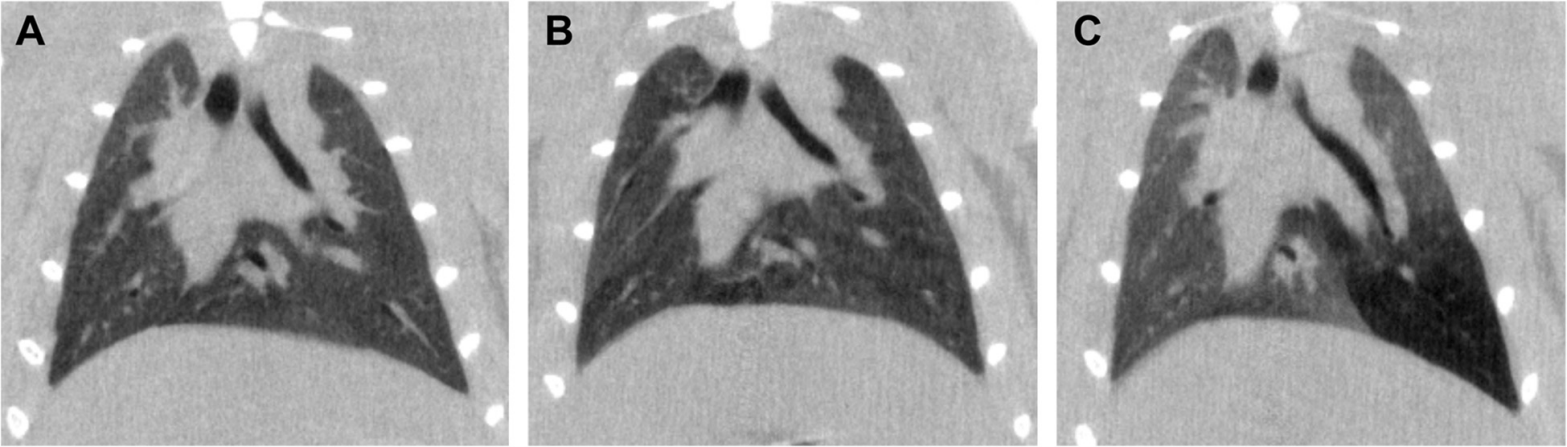
**Unfiltered coronal slices from 3D images of A) a control rat, B) a full-lung-dose rat, and C) a single-lobe-dose rat.** Only subtle differences between the full-lung-dose and control rats are evident; however, the distal region of the left lobe of the single-lobe-dose rat has considerably lower signal intensity, indicative of tissue destruction and/or air trapping characteristic of emphysematous disease.

Figure 2 shows a representative unfiltered coronal slice from a rat in each dose group: panel A is a control rat, panel B is a full-lung-dose rat, and panel C is a partial-lung-dose rat (the dose was delivered to the distal portion of the left lobe). The mean HU value (± standard deviation) of the control group was-544 ± 58 HU and the mean of the full-lung dose group was−594 ± 38 HU. In spite of marginally lower HU values that would be expected from emphysematous disease, an analysis of variance showed that the two groups were indeed not statistically different (p = 0.26). On the other hand, the partial-lung-dose rats showed a bimodal distribution of HU values because the diseased regions of the lung were distinct from the healthy regions. An example of this can be seen in the lower portion of the left lobe of the partial-lung-dosed rat (panel C) where the signal intensity is substantially lower than the rest of the lung (by ≈ 200 HU)–indicative of severe tissue destruction and air trapping, which are characteristics of emphysematous lungs [34]. Based on the overall HU measurements, we presume that the full-lung-dose rats developed a mild–and difficult to distinguish–emphysematous disease while the single-lobe-dose rats developed a more severe, albeit localized, disease.

### Octree decomposition tests

#### Threshold

The starting octree decomposition threshold level determined from the standard deviations of the control rats (Section Threshold range) was 60 ± 1 HU; therefore, 60 HU was used as the initial threshold level. Thus, the other threshold levels tested were 20, 40, 80, and 120 HU. Octree decompositions were performed using the different threshold levels on the three control rats, and variograms were compared using a KW rank sum test. The threshold level that showed the least difference among the control rats was 40 HU (p = 0.12), with 60 HU also showing no significant differences (p = 0.09). The 20, 80, and 120 HU thresholds did have significant differences among the controls (p = 0.02, p < 0.0001, and p < 0.0001, respectively). Based on this result, we chose to use the threshold level of 40 HU for all subsequent octree decompositions (unless otherwise noted).

#### Image filtering

Results of applying the different filters showed that the variograms from the unfiltered image and the image with the median filter were indistinct in an MWW test (p = 0.62). However, the relatively noisy unfiltered image resulted in about 20% fewer 8 × 8 × 8 boxes than the filtered image. This was in spite of a higher threshold that was used, 48 HU, based on the unfiltered image’s standard deviation. The radius = 2 and radius = 4 Gaussian filter results also did not differ significantly from the unfiltered image (p = 0.41 and p = 0.48, respectively) or from the median-filtered image (p = 0.71 and p = 0.84, respectively). A KW test showed that the radius = 2 filter resulted in control group variograms that were not distinct (p = 0.16), but the radius = 4 variograms were (p = 0.0001). However, because of the blurring caused by Gaussian filters, the number of 8 × 8 × 8 boxes that resulted from the Gaussian-filtered images was about 30% higher than the median filtered image, presumably because vascular structures and edges were not well preserved. Threshold levels used for the radius = 2 and radius = 4 images were 37 HU and 39 HU, respectively, based on the post-filtering standard deviations.

#### Effect of downsampling

**Figure 3:**
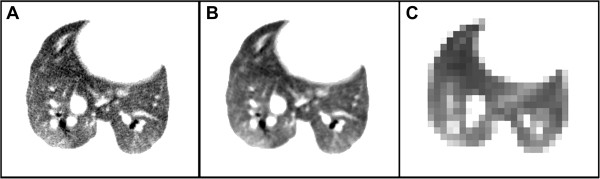
**A masked axial slice from an original CT image with examples of different filtering. A**: Unfiltered CT image. **B**: 3D median filter (5 pixel diameter). **C**: Downsampled by 8 × .

Figure 3 shows an example of an original image (panel A), the image with the five voxel diameter 3D median filter (panel B), and the same image downsampled to 1/8 resolution, or 64 × 64 × 64 pixels (panel C). The variograms made from the downsampled images of the three control rats were statistically different in a KW rank sum test (p < 0.0001). On the other hand, the variograms made from only the 8 × 8 × 8 boxes that came out of the octree decomposition were not (p = 0.12; see Section Threshold).

#### Translation and rotation

Results of image translation and rotation were compared for one rat. For the eight-pixel translation, variogram results were exactly identical to that of the original image, as expected. The four-pixel shift was not statistically distinct from the original image as confirmed by a MWW test (p = 0.96). In addition, the result of the rotation showed no significant change from the original (p = 0.56) or from the four-pixel shift (p = 0.59). Thus, we confirm that shifts or rotations to the image (i.e. alternative positions of the lung during imaging) do not result in significant changes to the resulting variograms.

### Octree decomposition

**Figure 4:**
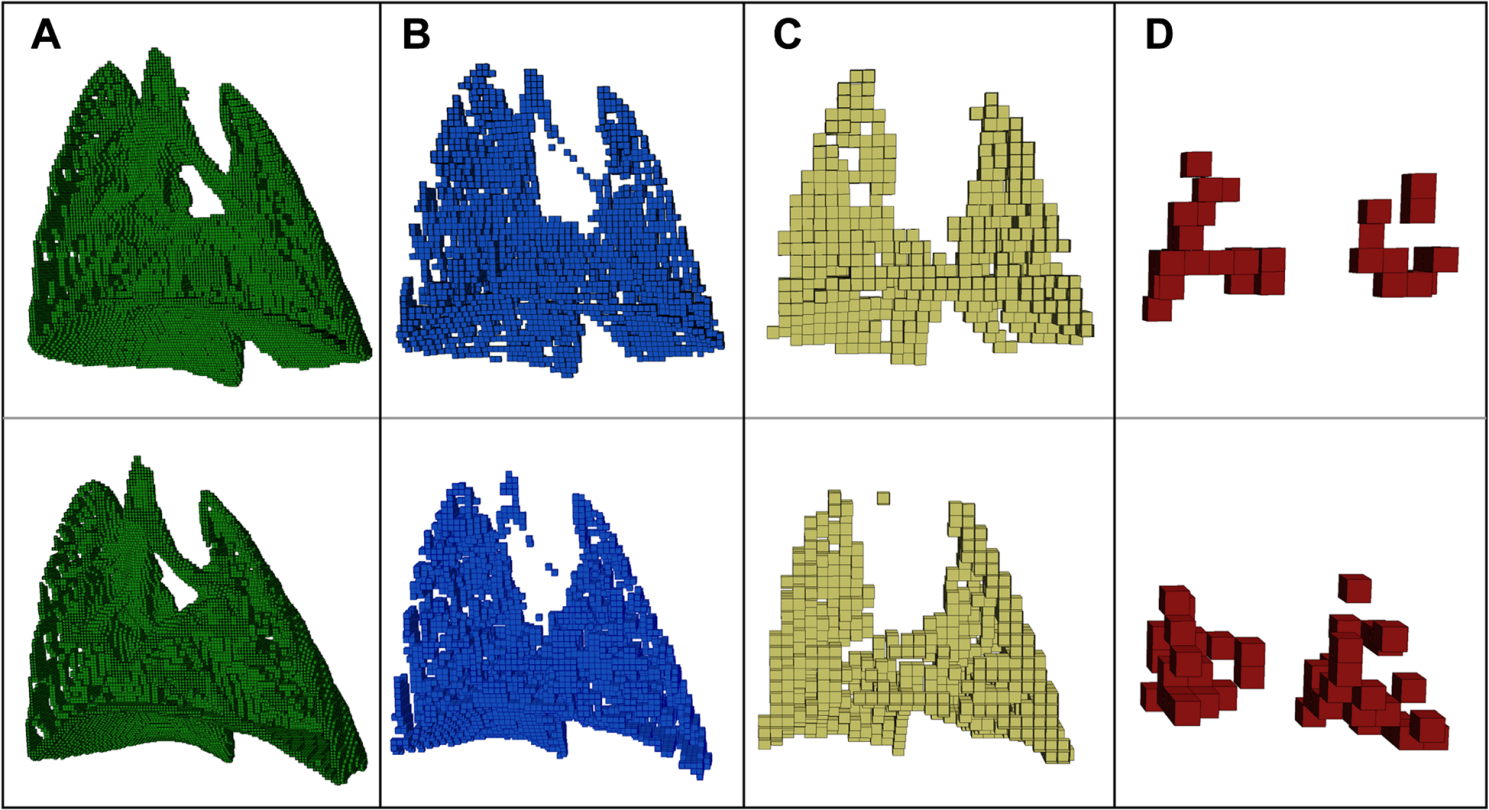
**Octree decomposition results from two rats.** The top row is a control rat and the bottom row is a single-lobe-dose rat (the same as those shown in Figure 2). Columns **A**, **B**, **C**, and **D** show the resulting 2 × 2 × 2, 4 × 4 × 4, 8 × 8 × 8, and 16 × 16 × 16 boxes, respectively.

Figure 4 shows the results of the octree decomposition on a control rat and on one with severe disease in the lower left lobe (see Figure 2C). Column A shows the 2 × 2 × 2 boxes, and column B shows the 4 × 4 × 4 boxes. These box sizes largely define the fine structures and edge details, including the conducting airways and vasculature. For this reason, we ignored the 2 × 2 × 2 and 4 × 4 × 4 boxes for the variogram analysis. Column C shows the 8 × 8 × 8 boxes and their relatively uniform distribution throughout the lung. Conversely, the less uniform distribution of the considerably fewer 16 × 16 × 16 boxes–the largest that resulted from octree decomposition–is shown in Column D. We point out, for example, that the lower left lobe of the treated rat (column D, bottom) has an approximately 2.5× higher density of 16 × 16 × 16 boxes than that of the control rat, indicating prevalence of localized homogeneous CT signal intensity.

**Figure 5:**
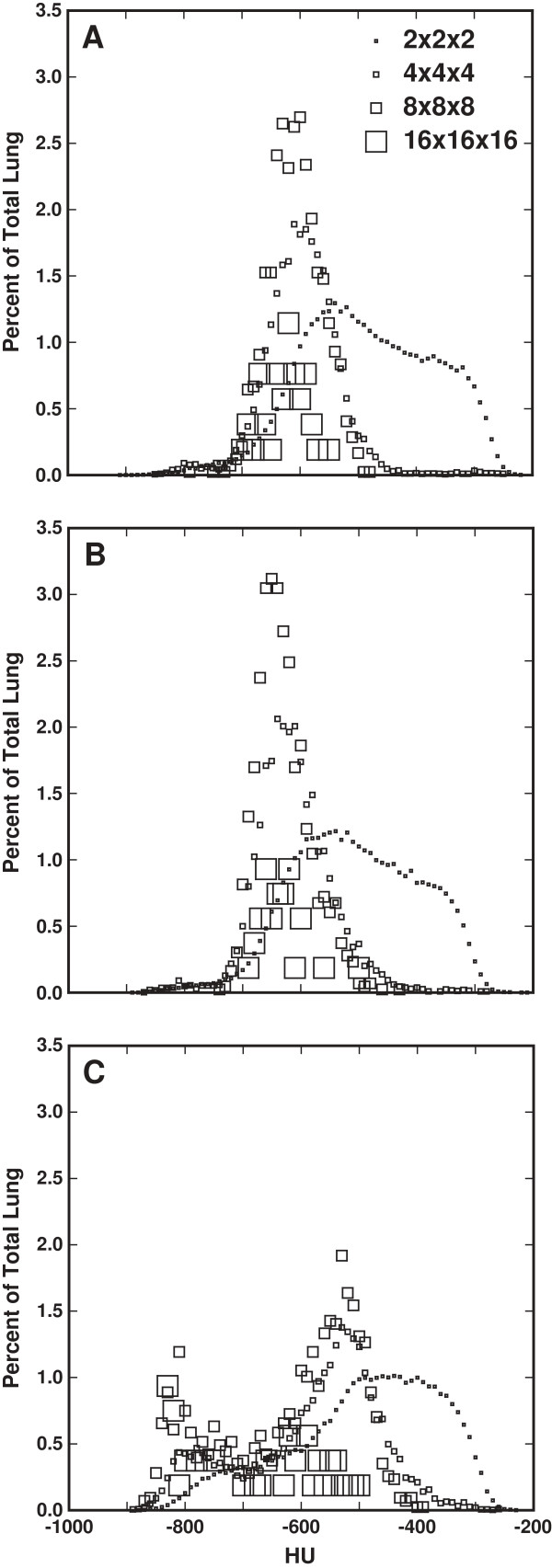
**Octree decomposition histograms showing the relationship between box size, HU, and percentage of the lung by each box size at each HU level for three representative rats. A**: Control (mean = -599 HU), **B**: Full-lung dose (mean = -634 HU), **C**: Single-lobe dose (mean of dosed region = -801 HU, mean of undosed region = -530 HU).

Histograms of the octree decomposition for three rats are shown in Figure 5. The marker size corresponds to the box size. In the control and mild disease rats (panels A and B), the different sized boxes, other than the 2 × 2 × 2’s, are approximately Gaussian distributed about the mean HU value of the lung, whereas the single-lobe-dose rat (panel C) shows a bimodal distribution, indicative of at least one large region of the lung with considerably lower HU values. These histograms show what percentage of the lung at each HU intensity is defined by the different box sizes, but they do not convey any information about the spatial relationships of the boxes.

### Variograms

**Figure 6:**
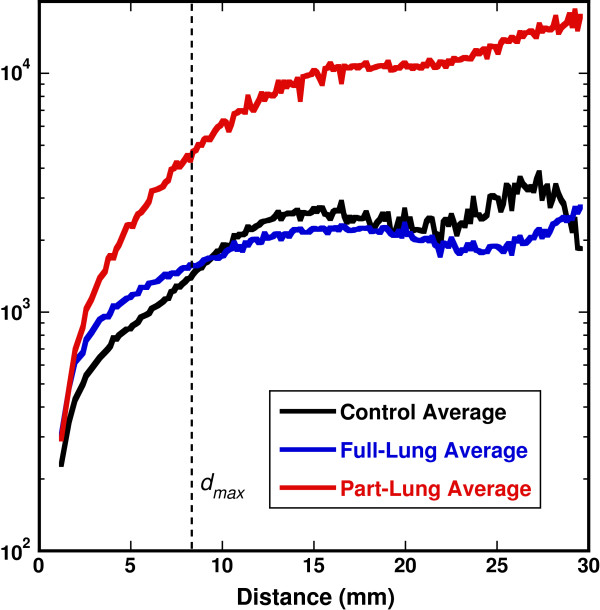
**Variograms averaged for each dose group.** The dashed line shows the extent of *d*_*max*_, the characteristic lung distance to which variograms were analyzed for heterogeneous variations. The lines get “noisier” at higher distances, indicative of increased spatial variation even within the each group.

In order to visualize the spatial relationships between the 8 × 8 × 8 boxes (and decomposed 16 × 16 × 16 boxes), variograms were constructed. Figure 6 shows the average variograms from the three different dose groups. The dashed line in Figure 6 denotes the range of *d*_*max*_ (see Section Variogram analysis). For *d* ≤ *d*_*max*_ a KW test showed that the control rats were statistically similar (p = 0.12), and a MWW test showed that the dose groups were each statistically distinct from the control group (p < 0.0001) and from one another (p < 0.0001).

### Emphysema index, coefficient of variation, and heterogeneity score

**Figure 7:**
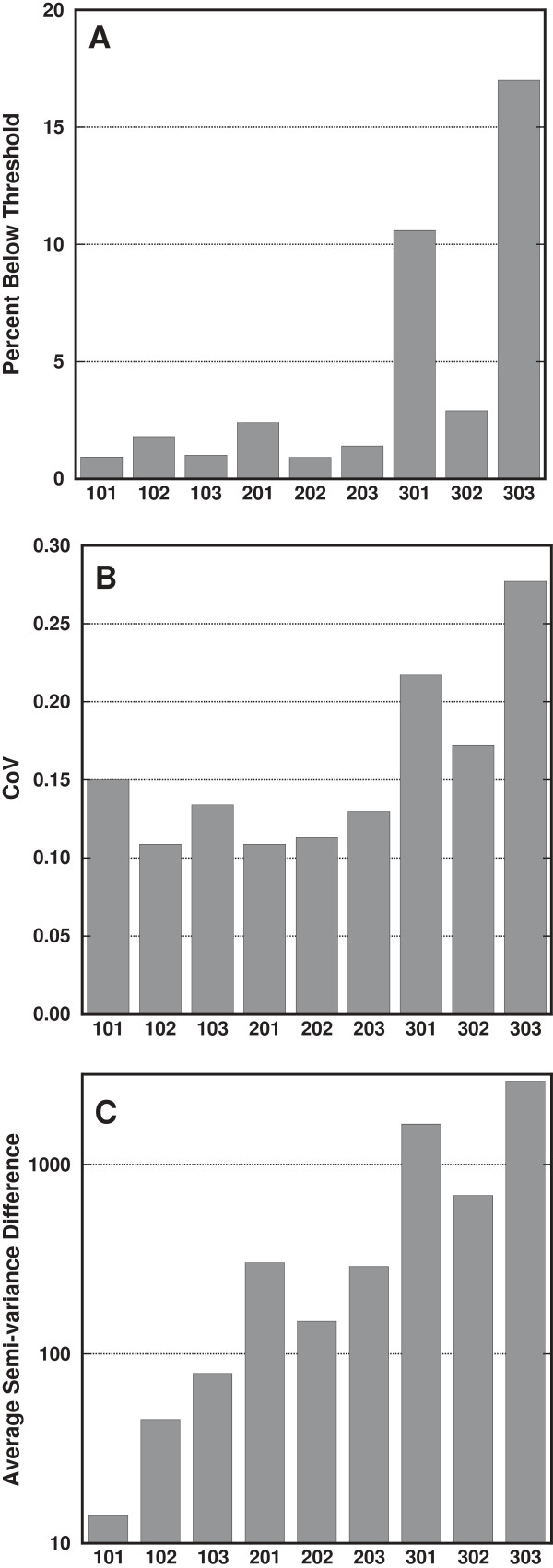
**Graphs comparing the different metrics for detecting emphysema. A**: Percentage of image pixels with HU values below two standard deviations from the mean of the control group. **B**: Coefficient of variation (CoV). **C**: The average distance of each individual rat’s variogram from the mean of the control group. The x-axis labels are animal numbers: 100s are controls, 200s are full-lung-dose, and 300s are single-lobe-dose. 301 and 302 received the dose in the right caudal lobe, and 303 received the dose in the distal region of the left lobe (Figure 2C).

Results of the emphysema index are shown in the Figure 7A. Although the number of rats is too small to reliably calculate sensitivity and specificity, the graph shows that there is considerable overlap between subjects in each dose group, likely indicating poor sensitivity and specificity. The CoV for each rat is shown in Figure 7B. Similarly, there is no distinction between the control and full-lung dose groups. The part-lung dose group had considerably higher CoV values, because the standard deviation was enlarged due to a bimodal (and non-Gaussian) distribution of HU values.

For comparison, we calculated the heterogeneity score Δ of each rat’s variance from that of the control group average; see Figure 7C. There is no overlap of Δ between dose groups (i.e. the highest Δ of the control group is lower than the lowest Δ of the full-lung group). This is not observed in Figure 7A or B, suggesting better sensitivity and specificity for Δ than the other metrics.

## Discussion

This work combined two different data analysis tools, octree decomposition and variograms, to study tissue heterogeneity in lung disease. We showed that this merged approach was better able to differentiate rats with mild emphysematous disease from the healthy control group than methods that relied on absolute HU values. The main criterion for octree decomposition was based on the standard deviation of HU values within an octree box. An advantage to this approach is that it avoids thresholding according to HU values, although sophisticated thresholding algorithms may be useful [35-37]; rather, it focuses only on heterogeneity-based signatures that may characterize disease [2]. We propose that a heterogeneity score Δ, the average distance of a rat’s variance from that of the control group average, may be useful to classify disease severity. Furthermore, to visualize the regions of the lung with the greatest heterogeneity, one could determine which boxes had the highest semi-variance within *d*_*max*_ and map them back to the original image. This would provide 3D information about the spatial distribution of lung tissue heterogeneity and, potentially, disease distribution.

Another approach to a disease metric might be that of fitting the data to an established variogram model, most of which describe an asymptotic rise in variance (i.e. variance becomes independent of distance indicating that spatial relationships become random) [23]. This is seen to some degree in our data (see Figure 6). However, within the range of *d* ≤ *d*_*max*_, we found that a power law model generally fit our data better than other models. This model typically describes fractal behavior [38]. Our initial investigations into this model showed potential promise at using fit parameters to distinguish dose groups; however, results lacked statistical significance, and the full-lung dose group tended to not fit this model as well as the other two groups.

There was no single variogram model that satisfactorily fit the all the data over the entire range of distances, because the complex geometries found in the lung result in some problems for variograms. In particular, the direct linear path between two regions of the lung separated by large distances often crosses non-lung tissue, such as the heart, which are essentially treated as holes or voids in the geometry. Furthermore, neighboring lobes generally do not interact physiologically except through the vascular and airway trees, which may only connect regions through many orders of branching. As pointed out by Keil et al. [25], one could go to extraordinary measures to take into account structural distances (the physiological distance at which different regions interact) versus Euclidian (straight line) distances used herein. To limit the problem in this study, we constrained the distance of variogram analysis to approximately half the characteristic diameter of the largest lobe. However, to better understand the inter-and intra-lobe variance relationships, the lung could be segmented into lobes (if the image is of sufficient resolution to discern lobar boundaries), and the decomposition/variogram process repeated on the segmented images. Though, by ignoring inter-lobar variances, this approach would likely not capture information about disease that was confined to a single lobe, particularly if the entire lobe was affected homogeneously.

We employed octree decomposition prior to generating the variograms. In doing this, we assumed that the emphysematous disease is generally slowly varying over space, and that the disease causes changes to homogeneity on the order of or less than the lobar length scale but greater than the octree box length scale [4,34]. This is consistent with what we previously observed using ^3^He MRI in the same disease model [26]. Without this assumption, variograms would have to be made directly from the raw images. This is possible but impractical, because the semi-variance computation time (and resulting file size) is proportional to the number of voxel pairs, which rises approximately as *n*^2^ (see Eq. 2). We measured the variogram computation time for 1078 octree-decomposed boxes from one rat to be 6.6 seconds (on a MacPro model 3.1), and we verified experimentally that the computation time indeed rose in proportion to the square of the number of voxels. Therefore, to create a variogram on the entire masked 3D image, which consisted of 1.48 × 10^6^ voxels (of lung tissue only), it would take us ≈ 1.25 × 10^7^ seconds, or about 5 months–with a resulting file size on the order of 20 GB. Therefore, octree decomposition dramatically reduces the computation time while focusing non-subjectively on regions of the lung that are of greatest interest. We note that the octree decomposition itself was performed in ~2 minutes.

Image noise can confound octree decomposition and affect resulting variograms. Results of image filtering tests indicated that noise reduction using an edge-preserving filter resulted in more 8 × 8 × 8 octree blocks without significantly affecting variogram results. An alternative to octree decomposition is downsampling, which is a quick and straightforward approach to reducing image noise and size. However, we verified that the octree decomposition approach performed much better at separating dose groups than simply downsampling the image and then calculating the semi-variance using every voxel. The octree decomposition assures that only the homogeneous regions of the lungs are singled out for comparison, whereas downsampling the image blurs together proximal voxels, including vasculature, airways, and lung boundaries irrespective of signal intensity or tissue type. Thus, the downsampling approach apparently causes a loss of information. The radius = 4 Gaussian filter had a result similar to downsampling.

One limitation of this pilot study was the small number of animals in each group, which did not allow the statistical evaluation of specificity and sensitivity. Therefore, follow-on work will be required to validate these results and establish specificity and sensitivity [2]. This might be accomplished in conjunction with pulmonary function tests, conventional morphometric measurements [39], and histological techniques particularly sensitive to early emphysematous changes [40]. Additional future work should evaluate the performance of this method on clinical CT images as well as test the effectiveness for distinguishing different diseases and disease models.

## Conclusion

Results of this pre-clinical study of elastase-treated rats suggests that automated octree decomposition and variogram analysis based on image heterogeneity may provide a non-objective and sensitive metric for characterizing emphysematous lung disease, even in early disease stages. The method outperformed conventional approaches that utilize thresholding and absolute HU values. This approach may be applicable to human datasets and other diseases.

## Competing interests

The authors have no conflicts of interest to disclose.

## Authors’ contributions

REJ designed the study, performed the experiments, analyzed the data, and drafted the manuscript. JPC developed Python code and participated in drafting the manuscript. Both authors read and approved the final manuscript.

## Pre-publication history

The pre-publication history for this paper can be accessed here:

http://www.biomedcentral.com/1471-2342/14/1/prepub
